# Routine, Routine, Routine: Sleep Regularity and its Association with Sleep Metrics in Professional Rugby Union Athletes

**DOI:** 10.1186/s40798-024-00709-5

**Published:** 2024-05-09

**Authors:** Angus Teece, Christopher Martyn Beaven, Haresh Suppiah, Christos K. Argus, Nicholas Gill, Matthew W. Driller

**Affiliations:** 1 Te Huataki Waiora School of Health, University of Waikato, Hamilton, New Zealand; 2 Chiefs Rugby Club, Hamilton, New Zealand; 3 Sport, Performance, and Nutrition Research Group, School of Allied Health, Human Services and Sport, La Trobe University, Melbourne, Australia; 4 New Zealand Rugby Union, Wellington, New Zealand

**Keywords:** Sleep Hygiene, SRI, Sleep Variation, Sleep Behavior, Sleep Efficiency

## Abstract

**Background:**

Maintaining a consistent sleep and wake time is often reported as a key component of circadian rhythmicity and quality sleep. However, the impact of sleep onset and offset time variability on overall sleep outcomes are underreported in elite athlete populations. This study investigated the relationship between sleep onset and offset time variability using the sleep regularity index (SRI) and measures of sleep and well-being in professional rugby union athletes. Twenty-three professional male rugby union athletes (mean ± SD, age: 23 ± 3 y) underwent sleep monitoring via wrist actigraphy for three weeks during a pre-season phase of training and completed a daily wellness questionnaire. Median SRI was calculated and used to stratify the trainees into two quantile groups: >76.4 SRI (Regular, *n* = 11) and < 76.4 SRI (Irregular, *n* = 12).

**Results:**

The regular sleep group showed significantly longer total sleep duration (*p* = 0.02, *d* = 0.97) compared to the irregular group (7:42 ± 0:29 vs. 7:18 ± 0:20 h: min per night, respectively). Furthermore, while not statistically significant, the regular sleep group showed greater sleep efficiency and less wake episodes compared to irregular sleepers, as demonstrated by *moderate* effect sizes (*d* = 0.71 and 0.69, respectively).

**Conclusions:**

The results from this study indicate that minimizing variability in sleep onset and offset time is beneficial for increasing sleep duration and may improve sleep efficiency during pre-season training in elite male rugby union athletes. This study provides evidence for the importance of including sleep-wake routines as a key component of sleep education interventions.

## Key Points

•When compared to irregular sleepers, regular sleepers demonstrated significantly longer total sleep duration (∼ 24 min per night) over a three-week period (> 9 h more of total sleep).

•Regular sleepers experienced fewer wake episode during the night, and had *moderately* higher sleep efficiency than irregular sleepers.

•The median sleep regularity index in this male, professional rugby cohort was 76.4, considerably lower than that reported in previous reports of elite team sport athletes.

•This study provides further support for the inclusion of promoting regular sleep-wake routines as a key component of sleep hygiene education.

## Background

Sleep is an important aspect that impacts recovery, skill execution, training, adaptation, and performance among elite-level athletes [1–3]. However, despite the importance of sleep, numerous studies have reported that elite athletes often experience insufficient quality and quantity of sleep [3, 4]. Due to the numerous challenges faced by elite athletes, including competition and training [5–7], travel [8], training volume [9] and other social factors [10], their sleep and wake routines may be highly variable and therefore are an area of interest. The sleep regularity index (SRI) has been suggested as a useful metric to inform consistency or variation in sleep and wake times [11]. The SRI calculates night-to-night shifts in sleep periods and is intended to reflect daily fluctuations in sleep and wake timings. The SRI produces a score out of 100, with 0 indicating no overlap between sleep-wake cycles from one day to the next, and 100 indicating an identical routine from one day to the next [11]. Currently, few studies evaluate the SRI in elite athlete populations, where keeping a consistent routine may be problematic.

Consistent sleep-wake cycles have been reported to be important for maximising the quality and quantity of sleep and are an important component of sleep hygiene education [12, 13]. Indeed, prior research has shown that shifting a sleep-wake cycle by as little as one hour can impact an individual’s quality and quantity of sleep for up to five days, which highlights the importance of consistency in sleep-wake cycles [14]. Furthermore, Lack et al. [15] reported that delaying sleep by approximately two hours on weekends resulted in longer sleep onset and reduced sleep duration on weekdays. As noted, sleep-wake cycles can be hindered within athlete populations by factors such as daily and weekly changes in training or competition schedules and travel [6, 7]. Sargent et al. [7] reported that elite swimmers reported delaying their sleep time the night before a rest day and delayed their wake time on the morning of rest days compared to training days. This pattern suggests that rest days may be seen as an opportunity for athletes to catch up on sleep lost and further supports that sleep-wake cycles may be highly variable among athlete populations.

To date, limited studies have evaluated the consistency or variation of sleep and wake time among elite athletes. Caia et al. [16] examined intraindividual sleep variability of junior and senior rugby league athletes and found that senior athletes showed less variability in sleep-wake patterns, time in bed, sleep duration, and sleep efficiency compared to junior athletes. Furthermore, Halson et al. [11] investigated the variation of sleep and wake times of 203 elite team sport athletes using the SRI. Halson and colleagues reported that athletes displayed a median SRI of 85.1 (out of 100). Athletes were divided into two groups, irregular sleepers who displayed an average SRI score of 76.5 out of 100, and regular sleepers who displayed an average SRI score of 90.1 out of 100. Regular sleepers displayed significantly greater sleep efficiency and less variability in total sleep time and sleep efficiency compared to irregular sleepers. However, results from this study found that regularity in sleep did not influence total sleep duration in this group of athletes across four different sports. Given the differences in schedules and routines between sports, further investigation of the SRI within a single sport is warranted.

The current investigation is the first study to examine sleep regularity in a cohort of professional male rugby union athletes, comparing SRI scores to metrics of sleep quality and quantity. The aims of this study were to (1) investigate SRI in a cohort of professional rugby union athletes over a three-week pre-season period, and; (2) compare sleep indices and measures of wellness between regular and irregular sleepers in professional rugby union athletes.

## Methods

### Participants

A total of 23 professional male rugby union athletes (mean ± SD; age, 23 ± 3 y; body mass, 104.9 ± 10.7 kg; stature, 187.0 ± 6.9 cm) from a New Zealand Super Rugby team volunteered to participate in the current study. Inclusion criteria included athletes being from the same professional rugby team and being free from any clinically diagnosed sleep disorder. Injured athletes were excluded from the study, and the study took place during the early pre-season phase of training (November– December 2019). Written informed consent was obtained from the participants, and ethical approval was obtained from an Institutional Research Ethics Committee (FEDU066/16) and complied with the Declaration of Helsinki for Human Research of 1974 (latest revision in 2000).

### Experimental Approach

An observational study design was used for the current investigation. Quantitative sleep and wellness assessments were collected throughout a three-week pre-season training phase. The relationship between qualitative sleep and wellness measures to sleep and wake variance via the use of the sleep regularity index (SRI) was examined across the study period. The three-week training period comprised of two speed sessions, four gym-based resistance training sessions, five conditioning sessions, and eight rugby-specific sessions spread across four training days each week. Each training day commenced at 8am with players arriving at the training facility. The first section of training took place between 9-11am which was either a unit specific session or a gym session each lasting an hour in duration. The second block of training which was team training commenced at 2:30pm and concluded approximately 4pm. The team’s professional strength and conditioning and coaching staff designed and monitored all training programs.

On field training load was assessed via GPS and was planned across the period of the study average weekly running load across the study period was 23.9 km + 3.3 km and 23.3 km = 3.1 km for the irregular and regular groups respectively (*p* = 0.74).

### Procedures

#### Sleep Monitoring

Quantitative sleep measures were collected using wrist actigraphy devices (ReadiBand™, Fatigue Science, Vancouver, BC, Canada) sampling at 16 Hz throughout the three-week training block. Athletes were required to wear wrist actigraphy on either wrist [17] at all times except during contact training throughout the study. Additionally, athletes were instructed to maintain their regular sleep routine throughout the study. All athletes slept in their home sleep environments for the duration of the study period. At the beginning of each day, the actigraphy data were wirelessly downloaded to online software (ReadiBand™, Fatigue Science, Vancouver, BC, Canada), which automatically scored the raw data. The data collected by the wrist actigraph was translated into sleep-wake indices, including total sleep time (TST), total time in bed (TTB), sleep efficiency (SE% - calculated by dividing TST by TTB), sleep latency (SL), wakefulness after sleep onset (WASO), wake episodes (WE), sleep onset time (SOT), and wake time (WT) for each sleep period. The ReadiBand™ has been validated against polysomnography (PSG) and has been deemed acceptable, with accuracy levels of ∼ 93% reported previously [18]. The ReadiBand has also been shown to be comparable to the manually-scored actigraph [19]. Additionally, the interdevice reliability of the ReadiBand™ has shown high levels of agreement (ICC ≥ 0.90) for the sleep monitoring device used in the current study [20].

### Wellness Assessment

A wellness questionnaire based on previous work by Hooper and Mackinnon [21] was completed on the morning of all training days of the data collection period. The wellness questionnaire was specifically designed for this study and asked athletes to rate their fatigue, general muscle soreness, stress, and sleep quality on a 1 to 7 Likert-type scale. On the scale, 1 represented a low score (e.g., positive result) and 7 represented a high score (e.g., negative result) for each measure. An overall wellness score for each athlete was calculated by combining responses to each question. Scores ranged from 4 to 28, with a lower score being considered to represent greater overall wellness. Research from our laboratory has shown that the wellness questionnaire displays acceptable day-to-day reliability with an ICC of 0.73 and a typical error (TE) of 1.63%.

### Sleep Questionnaires

The Athlete Sleep Behavior Questionnaire (ASBQ) and the Pittsburgh Sleep Quality Index (PSQI) were collected via Google Forms (Google LLC, Mountain View, CA, USA) at the conclusion of data collection to assess various aspects of sleep behaviour across the data collection period. The ASBQ is an 18-item sleep questionnaire which contains questions about sleep habits and behaviours thought to be areas of concern amongst athlete populations [22]. The survey asks participants how frequently they engaged (never, rarely, sometimes, frequently, always) in specific behaviours that have been considered maladaptive over the past month. The response to each question is weighted on a scale of 1–5 (never = 1, rarely = 2, sometimes = 3, frequently = 4, always = 5), and all questions are summed to produce an ASBQ global score. Global scores range from 18 to 90, with a higher score indicating poor sleep behaviours. The PSQI is a 19-item self-rated questionnaire intended to assess sleep disturbances and quality over the past month [23]. The questionnaire is separated into 7 components (subjective sleep quality, sleep latency, sleep duration, habitual sleep efficiency, sleep disturbances, use of sleep medications and daytime dysfunction) and each component is weighed on a scale of 0–3. A higher score indicates a poor habit or behaviour in that component. All components are summed to produce a PSQI global score. Global scores range from 0 to 21, with a higher score indicating worse sleep behaviours.

### Sleep Regularity Index

The sleep regularity index (SRI) is a metric that assesses night-to-night shifts in sleep and wake cycles by accounting for changes in sleep onset and wake times. The SRI assesses the likelihood that an individual’s sleep-wake cycle matches from one day to the next, which was then aggregated over the study period to provide a total SRI score. An SRI score of 100 indicates that the sleep-wake cycle was identical between one day and the next. Conversely, an SRI score of 0 indicates no overlap between sleep-wake cycles from one day to the next. SRI values of each athlete were determined using binary sleep-wake data. Each sleep onset and wake time was derived from wrist actigraphy and converted into UNIX time, representing the time and day. Each UNIX time was coded as either ‘1’ for sleep time or ‘0’ for wake time. SRI calculations were conducted using the R program (Rstudio) using the *sleepreg* package [24].

As described by Windred et al. [24], SRI scores were calculated using the following equation:

$$ SRI = - 100 + 200\left( {1 - \frac{1}{{{N_v}}}\sum\nolimits_{i = 1}^N {\left| {{s_i} - {s_{i + C}}} \right|} } \right) $$

Sleep-wake state is represented by $$ {s}_{i}=1$$ for wake, $$ {s}_{i}=0$$ for sleep, and $$ {s}_{i}=NA$$ represents excluded epochs. Number of valid epoch-by-epoch comparisons is represented by $$ {N}_{v}$$, which includes all comparisons where $$ {s}_{i}\ne NA$$ and $$ {s}_{i+c}\ne NA$$. Where $$ {s}_{i}=NA$$ or $$ {s}_{i+c}= NA$$, $$ \left|{s}_{i}-{s}_{i+c}\right|=0$$. Subscript $$ i$$ represents each epoch from recording start to 24 h prior to recording end, such that at:

$$ i=1, $$ $$ {t}_{1}=0$$

$$ i=2, $$ $$ {t}_{2}=E$$

$$ i=3, $$ $$ {t}_{3}=2E$$

$$ i=C, $$ $$ {t}_{C}=E\left(C-1\right)=24$$

$$ i=N, $$ $$ {t}_{N}=E(N-1)={t}_{max}-24$$

Time is represented by $$ {t}_{i}$$, epoch length is represented by $$ E$$, recording length is represented by $$ {t}_{max}$$, and number of epochs within one 24-h interval is represented by $$ C$$. All time values are in hours.

Following the calculation of each individual’s SRI score, each athlete was classified as either regular (*n* = 11) or irregular (*n* = 12) sleepers. Specifically, athletes were classified as Regular sleepers if they were above the median (> 76.4) of the cohort based on their SRI scores, while participants who were below the median (< 76.4) based on their SRI score, were considered Irregular sleepers.

### Statistical Analyses

All descriptive statistics are reported as means ± SD unless otherwise stated. Statistical analysis was performed using Statistical Package for Social Sciences V27.0 (IBM Corporation; Chicago, IL, USA) with statistical significance set at *p* ≤ 0.05 for all analyses. Data of sleep regularity, sleep measures, wellness assessment, and sleep questionnaires were pooled, and Pearson’s correlations (*r*) were used to determine if any relationships were present between sleep, markers of wellness, and sleep questionnaire global scores. Correlations were interpreted using thresholds of *r* < 0.1, *trivial*; 0.1–0.3, *small*; 0.3–0.5, *moderate*; 0.5–0.7, *large*; 0.7–0.9, *very large*; and 0.9-1.0, *almost perfect*. A comparison between regular and irregular sleepers was made for sleep indices, wellness scores, and global sleep questionnaire scores usingin dependent samples *t*-tests. There were no outliers in the data as assessed by inspection of a boxplot. Each variable was normally distributed as assessed by Shapiro-Wilk’s test (*p* > 0.05). Effect-size statistics were calculated using Cohen’s *d* and interpreted using thresholds of 0.2, 0.5, and 0.8 for *small, moderate*, and *large*, respectively. An effect size of -0.2 > 0 < 0.2 was considered *trivial*, and the effect was deemed *unclear* if the 95% confidence interval overlapped the thresholds for both positive and negative effects [25].

## Results

**Fig. 1:**
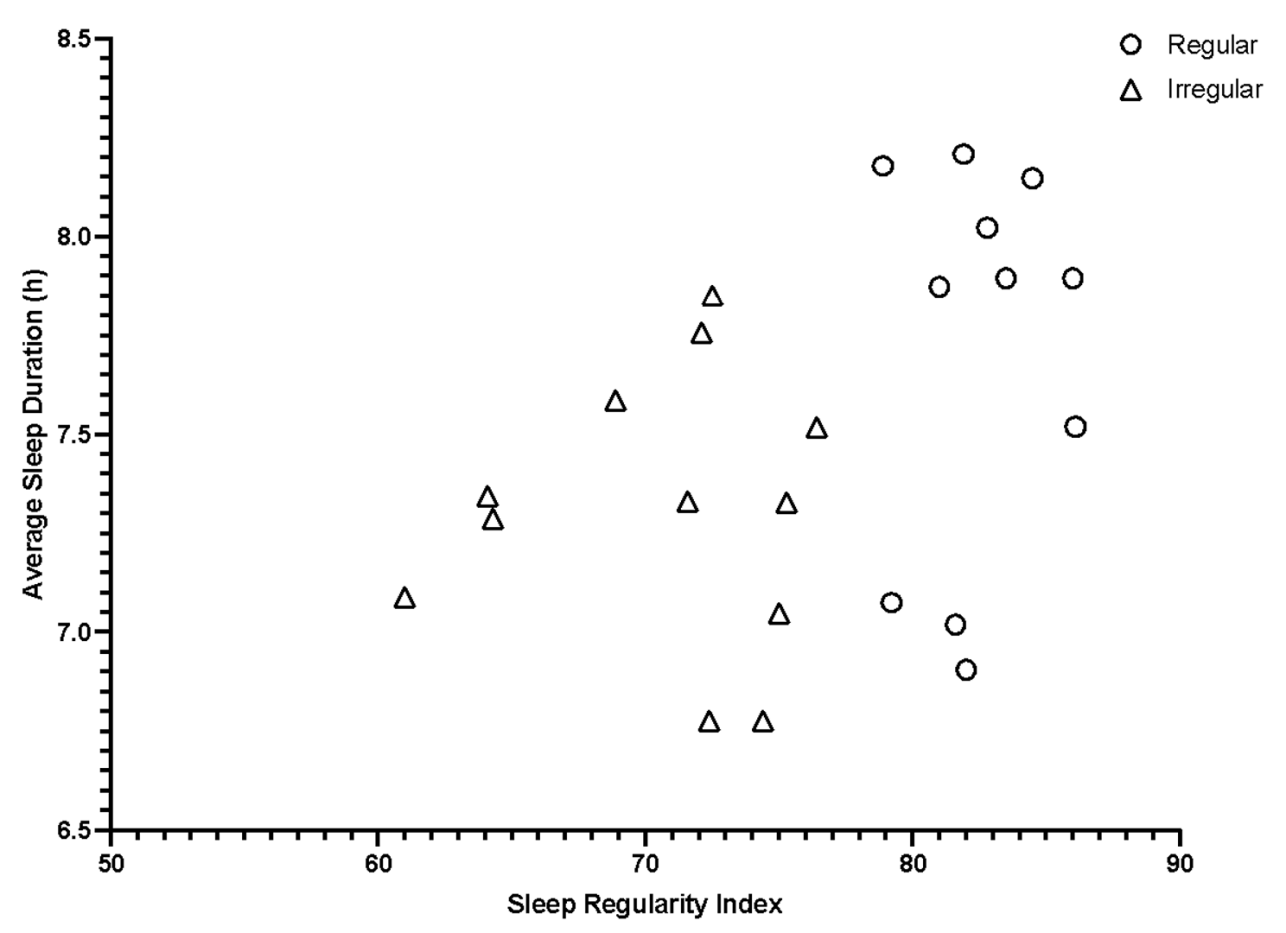
The relationship between sleep regularity index score and average sleep duration for all participants split into Regular (*n* = 11) and Irregular (*n* = 12) sleepers in elite rugby union athletes

Descriptive analysis of sleep regularity scores across the cohort revealed that the highest SRI score was 86.1, with the cohort median being 76.4, and the lowest SRI value being 61.0. Furthermore, Pearson’s correlation analysis revealed a *moderate* correlation between SRI and average *(r* = 0.40; Fig. 1) and total sleep duration (*r* = 0.43), while all other measures of sleep and wellness showed *small* to *trivial* correlations with SRI.

Independent sample t-tests revealed a significant difference in average sleep duration per night (*p* = 0.03) and total sleep duration over the 3-week period (*p* = 0.02) in favour of the Regular sleeping group, as shown in Table 1. The results showed that Regular sleepers achieved ∼ 24 min longer sleep duration on average per night and ∼ 9 h more total sleep across the 3-week period. No significant differences (*p* > 0.05) were observed between Regular and Irregular sleepers for sleep latency, wake episodes, WASO, and sleep efficiency. The effect size analysis revealed that Regular sleepers displayed *moderately* (*d* ± 95% CI = 0.71 ± 0.83) fewer wake episodes per night and a *moderately* higher (*d* = 0.69 ± 0.85) sleep efficiency compared with Irregular sleepers.

**Table 1:** Differences in sleep indices assessed via wrist actigraphy (mean ± SD) between Regular (*n* = 11) and Irregular (*n* = 12) sleepers in elite rugby union athletes, including *p*-values and effect size comparison (with 95% confidence intervals) between groups

Measure	Mean ± SD		*p*-value	Effect size (d ± 95% CI)
	Regular	Irregular		
Average Sleep Time per night (h: min)	7:42 ± 0:29	7:18 ± 0:20	0.03*	0.89 ± 0.85 ***large***
Total Sleep Duration over 3 weeks (h: min)	169:28 ± 10:48	160:07 ± 7:19	0.02*	0.97 ± 0.85 ***large***
Average Time in Bed per night (h:min)	9:09 ± 0:16	8:28 ± 0:25	< 0.01	1.84 ±0.89 ***large***
Average Sleep Onset Time (time of day)	22:37 + 0:47	23:18 + 0:56	0.08	0.75 + 0.86 *unclear*
Average Wake Time (time of day)	7:11 + 0:29	6:58 + 0:42	0.26	0.46 + 0.85 *unclear*
Sleep Latency (min)	28.9 ± 16.8	32.9 ± 11.2	0.50	0.27 ± 0.85 *unclear*
Wake Episodes per night (no.)	2.9 ± 1.0	3.8 ± 1.3	0.09	0.71 ± 0.83 ***moderate***
Wake after sleep onset (h: min)	0:28 ± 0:12	0:36 ± 0:15	0.17	0.57 ± 0.83 *unclear*
Sleep Efficiency (%)	85.2 ± 3.6	83.1 ± 2.2	0.09	0.69 ± 0.85 ***moderate***

*indicates a significant difference between groups (*p* < 0.05)

The results of the independent sample *t*-tests revealed no significant differences (*p* > 0.05) between Regular and Irregular sleepers for any of the wellness measures (fatigue, muscle soreness, stress, sleep quality, and overall wellness), as shown in Table 2. Additionally, no significant differences (*p* > 0.05) were observed between Regular and Irregular sleepers for global scores derived from the ASBQ (*p* = 0.65) and the PSQI (*p* = 0.88). However, inspection of individual questions of the ASBQ revealed significant differences (*p* < 0.05) with Irregular sleepers reporting higher (poorer) scores for Q5, “*I go to bed at different times each night (more than ± 1 hour variation)*” and Q12, “*I wake to go to the bathroom more than once per night*”.

**Table 2:** Differences in wellness measures as assessed by a wellness questionnaire (mean ± SD) for the Regular (*n* = 11) and Irregular (*n* = 12) sleepers in elite rugby union athletes, including *p*-values and effect size comparison (with 95% confidence intervals) between groups. AU = arbitrary units

	Mean ± SD		*p*-value	Effect Size (d ± 95% CI)
	Regular	Irregular		
Fatigue score (AU)	4.1 ± 0.5	4.1 ± 0.7	0.96	0.02 ± 0.84 *unclear*
Muscle Soreness (AU)	3.8 ± 0.8	3.8 ± 0.9	0.81	0.10 ± 0.83 *unclear*
Stress (AU)	4.7 ± 0.9	4.4 ± 0.8	0.37	0.36 ± 0.84 *unclear*
Sleep Quality (Self-reported)	4.3 ± 0.9	4.2 ± 0.8	0.82	0.09 ± 0.83 *unclear*
Overall Wellness (AU)	17.0 ± 2.3	16.7 ± 3.0	0.75	0.13 ± 0.83 *unclear*
ASBQ (AU)	41.1 ± 4.0	42.0 ± 5.4	0.65	0.18 ± 0.83 *unclear*
PSQI (AU)	5.2 ± 1.6	5.4 ± 2.7	0.88	0.06 ± 0.83 *unclear*

## Discussion

The current investigation aimed to compare measures of sleep and well-being across a pre-season phase of training in elite male professional rugby union athletes who displayed regular sleep onset and offset times against those who displayed irregular sleep onset and offset times. Key findings highlighted that maintaining a more regular sleep onset and offset time across a pre-season period resulted in ∼ 24 min longer sleep duration on average per night, and ∼ 9 h more total sleep across the three-week period compared to athletes who displayed irregular sleep patterns. Increased sleep duration appears to be important for improving reaction time, stress hormone suppression [26], aerobic performance, and body composition [2, 27] in the pre-season in elite rugby union athletes. Therefore, the finding that maintaining regular sleep onset and offset routines may be associated with increased sleep duration is a novel and important finding for team sport athletes attempting to maximise their performance and physiological adaptations. Furthermore, although not statistically significant, we observed that the more regular sleepers showed *moderately* lower wake episodes per night and *moderately* higher sleep efficiency when compared with their counterparts who were considered irregular sleepers.

To the authors’ knowledge, this study is the first to show a relationship between SRI and total sleep time in professional rugby athletes. Since SRI is a relatively new metric of sleep consistency, there are limited studies reporting this within athlete populations. The findings of the current investigation of the regular group displaying increased sleep duration are in contrast with previous studies investigating SRI. Indeed, Halson et al. [11] investigated SRI among a cohort of 203 elite team sport athletes across four sports and found that SRI had no impact on total sleep time. It is important to note that the investigation by Halson and colleagues required athletes to have their sleep monitored for a minimum of seven nights to be included in the study. Therefore, the relatively shorter monitoring period (average of ∼ 10 nights) combined with the heterogenous sample (multiple sports) may have influenced the ability to discover differences in sleep duration between regular and irregular sleepers. Our median SRI score (76.4) was also notably lower than the score of 85.1 reported by Halson et al. [11]., perhaps due to the same reasons stated above. Furthermore, Phillips et al. [28] investigated the SRI in an age-matched university cohort across a 30-day period and found no differences in average sleep duration between regular and irregular sleepers (7:27 and 7:16 h: min, respectively). Although it is unclear why Phillips and colleagues did not observe any differences in total sleep time between regular and irregular sleepers, the research team suggested that due to the number of students living on campus, they displayed polyphasic sleep schedules and therefore may have maintained sleep durations irrespective of their SRI. It is important to note that within the current investigation, while the data was not collected, it is possible that the athletes did not have the opportunity to nap during the day given their training schedules, and therefore sleep duration was achieved only through night-time sleep, which may in part explain the differences observed between the current investigation and Phillips et al. [28].

While speculative, the positive effects observed for the duration of sleep may be due to better regularity in sleep onset and offset times supporting the entrainment of circadian rhythms [29]. Circadian rhythms have been shown to affect body temperature [30], melatonin secretion, and sleep propensity [31], which are all important drivers of sleep [32]. Prior research suggests that high sleep and wake time variability may lead to circadian rhythm disturbances [33]. Circadian rhythm disruptions result in sleep disturbances caused by advancing or preventing sleep, including insomnia, and decreased sleep duration [34, 35]. Conversely, regularity in sleep timing may support circadian rhythms in remaining oriented to an individual’s daily environment and routine [29]. It is plausible that maintaining regularity in sleep onset and offset times may assist in optimising circadian rhythms, subsequently influencing sleep duration, as seen in the current investigation.

Whilst not statistically significant, we observed a *moderate* increase in sleep efficiency in favour of regular sleepers. Similar findings have been observed by Halson et al. [11], who found that regular sleepers displayed significantly greater sleep efficiency than irregular sleepers. Halson et al. [11] reported that bedtime (the time at which the individual attempted to start sleep), a consistent sleep onset time (the time at which an individual first fell asleep), and sleep offset time were important factors that contributed to improved sleep efficiency among athletes. Furthermore, the number of wake episodes per night may have influenced sleep efficiency in the current investigation. We observed that irregular sleepers displayed more wake episodes than regular sleepers, as demonstrated by a *moderate* effect size difference between groups. This more frequent waking coincided with higher reporting of waking up greater than once per night to go to the bathroom compared to regular sleepers. In fact, since sleep efficiency is calculated as a ratio of total sleep time divided by time in bed, a loss of sleep time due to increased wake episodes will decrease sleep efficiency and may, in part, explain the results of the current investigation. Interestingly, aside from the SRI in the current study, regular sleepers self-reported less frequently going to bed at different times each night in Q5 of the ASBQ, suggesting that athletes may already know how consistent their routines are without undergoing sleep monitoring. Therefore, practitioners may be able to ascertain sleep regularity via subjective responses, without the need for objective monitoring.

Given the applied nature of the present study and the difficulties of conducting research on professional athletes, we acknowledge several limitations. First, this study was designed to create minimal impact on the training and performance of each athlete, meaning that we could only employ an observational study design with retrospective analysis. Furthermore, SRI only accounts for night-time sleep duration, and therefore daytime naps (data not collected) were not included in the total sleep duration, which may have influenced the total sleep duration and pressure individuals experienced over a 24-hour period. Additionally, the current investigation was over a three-week period, and therefore, it only assessed SRI during a relatively short period of an athlete’s overall season. It should be considered that this study was conducted within a period where no competition was present; therefore, it may have missed important factors such as travel and match day influences that can affect sleep onset and wake time variability during a competition season [5]. Lastly, this investigation was performed on male rugby union athletes, as this was the only professional rugby cohort available to the researchers. Whether the same findings can be generalised to elite female rugby athletes is yet to be explored. Future research should investigate an in-season training phase to assess the impact of various other factors on SRI and sleep metrics and include professional female rugby union athletes to determine whether the same relationships between SRI and sleep metrics apply to them.

## Conclusion

The current investigation is the first to show a relationship between sleep regularity index and sleep metrics in an elite male rugby union environment during a pre-season phase of training. The results suggest that maintaining a more consistent sleep onset and offset time is associated with improved sleep duration and trends towards higher sleep efficiency and less wake episodes per night (both associated with *moderate* effect sizes compared to irregular sleepers). This study provides further evidence for including sleep-wake routines as a key component of sleep monitoring and educational sessions with athletes during periods in which the quality and quantity of sleep can often be compromised.

## Data Availability

The datasets used and/or analysed during the current study are available from the corresponding author on reasonable request.

## Abbreviations

ASBQ: Athlete sleep behavior questionnaire
PSG: Polysomnography
PSQI: Pittsburgh sleep quality index
SD: Standard deviation
SE%: Sleep efficiency percentage
SL: Sleep latency
SOT: Sleep onset time
SRI: Sleep Regularity Index
TST: Total sleep time
TTB: Total time in bed
WASO: Wake after sleep onset
WE: Wake episodes
WT: Wake time
